# Evaluation of the diagnostic accuracy of an affordable rapid diagnostic test for African Swine Fever antigen detection in Lao People’s Democratic Republic

**DOI:** 10.1016/j.jviromet.2020.113975

**Published:** 2020-12

**Authors:** Nina Matsumoto, Jarunee Siengsanan-Lamont, Laurence J. Gleeson, Bounlom Douangngeun, Watthana Theppangna, Syseng Khounsy, Phouvong Phommachanh, Tariq Halasa, Russell D. Bush, Stuart D. Blacksell

**Affiliations:** a Sydney School of Veterinary Science, University of Sydney, Private Bag 4003, Narellan, NSW, 2567, Australia; b Mahidol-Oxford Tropical Medicine Research Unit, Faculty of Tropical Medicine, Mahidol University, 420/6 Rajvithi Rd., Bangkok, 10400, Thailand; c National Animal Health Laboratory, Department of Livestock and Fisheries, Ministry of Agriculture and Forestry, Souphanouvong Avenue, Sikhottabong District, PO. Box 6644, Vientiane, Lao Democratic People’s Republic; d Department of Veterinary and Animal Sciences, Faculty of Health and Medical Sciences, University of Copenhagen, 1870, Frederiksberg C, Denmark; e Centre for Tropical Medicine & Global Health, Nuffield Department of Medicine, University of Oxford, Oxford, United Kingdom; f Lao-Oxford-Mahosot Hospital-Wellcome Trust Research Unit (LOMWRU), Mahosot Hospital, Vientiane, Lao Democratic People’s Republic

**Keywords:** African Swine Fever, Rapid diagnostic test, Lao PDR, Diagnostic accuracy, Polymerase chain reaction

## Abstract

African Swine Fever (ASF) is a transboundary animal disease of pigs and wild suids that appeared in Lao People's Democratic Republic (Lao PDR) in mid-2019, having spread across China and Vietnam in the months prior. Despite the scale of the Asian ASF pandemic and the availability of pen-side rapid diagnostic tests (RDT) on the market, few locally produced and easily available ASF RDTs have been evaluated for diagnostic accuracy. In this study, an ASF antigen detection RDT from Shenzhen Lvshiyuan Biotechnology Co. Ltd was evaluated using clinical field samples submitted to the National Animal Health Laboratory (NAHL) from ASF suspect cases between June and December 2019 in Lao PDR. Positive (n = 57) and negative (n = 50) samples of whole blood, serum and haemolysed serum were assessed by RDT and PCR, with the latter used as the gold standard reference comparator. Overall the RDT had a diagnostic sensitivity (DSe) of 65 %, 95 % CI [51–77] and diagnostic specificity (DSp) of 76 %, 95 % CI [62–87]. The RDT demonstrated improved performance on samples with lower PCR cycle threshold (*ct*) values with each additional cycle reducing the odds of the RDT returning a positive by 17 % relative to the previous cycle, 95 % CI [8 %–28 %] (P < 0.01). While this test shows promise for field application, complete validation of diagnostic accuracy requires a larger sample size.

African Swine Fever (ASF) is a DNA virus in the family *Asfarviridae*, genus *Asfivirus*. ASF is a viral transboundary animal disease affecting suids, easily transmitted by fomites and carried by soft ticks as a vector in east African and Iberian contexts (Sánchez‐Vizcaíno et al., 2019). The ability of Southeast Asian soft ticks to carry ASF as a vector is as yet unstudied. ASF is moderately contagious but has up to 100 % mortality rates in affected herds (Sánchez‐Vizcaíno et al., 2019). The virus can survive in the environment for extended periods and is present in all tissues and secretions of infected animals (Davies et al., 2017). Lao People's Democratic Republic (Lao PDR) recorded its first outbreak of ASF in June 2019 (FAO, 2020) where pig farms experienced peracute and acute syndromes during this outbreak. Due to the dynamic nature of pig movements in southeast Asia (Leslie et al., 2015; Poolkhet et al., 2019), ASF necessitates effective outbreak control before farmers rush to sell their symptomatic and at-risk pigs at a lower price rather than face losing the asset entirely (Dione et al., 2016).

Broadly, ASF diagnostic assays fall into three categories: viral nucleic acid detection, antigen detection and antibody detection (Sánchez-Vizcaíno and Mur, 2013). The Lao Ministry of Agriculture and Forestry coordinates ASF diagnosis between sample collection at the field level and centralised testing at the National Animal Health Laboratory (NAHL) in Vientiane. The NAHL use real-time PCR (King et al., 2003) for the reference diagnosis of ASF. However, the use of PCR as a sole diagnostic puts considerable strain on resources at the laboratory and delays field-based investigations and disease control activities during an active outbreak. Furthermore, Laos lacks a formal sample submission system and relies on public buses to transport test samples at ambient temperatures which may affect sample quality and cause delays in diagnosis. Rapid diagnostic tests (RDT) have been developed for the diagnosis of ASF detection of viral antigen. A European test strip (INgezim ASF CROM Ag from Eurofins Technologies Ingenasa^1^) performed with diagnostic sensitivity (DSe) of 67.86 % and diagnostic specificity (DSp) of 97.98 % and is the only lateral flow assay to be evaluated at the time of publication (Sastre et al., 2016). Bionote Inc.^2^ recently brought the Anigen Rapid ASFV Ag rapid test to market which shows good agreement with the reference PCR, however full validation data is not yet available (M. Thacker, pers comm, 2020). Additionally, a number of similar format tests are being produced in China, but these are yet to be evaluated for diagnostic accuracy.

Measures of diagnostic accuracy like DSe and DSp allow operators to define a test’s ability to distinguish between a healthy animal and one that is affected by a disease of interest. In this study, we estimate the DSe and DSp of the Chinese-manufactured Shenzhen Lvshiyuan Biotechnology^3^ (SLB) ASF antigen detection RDT, which uses the colloidal gold immunochromatographic method. When a sample is placed in the testing well of the RDT, the virus will bind to colloidal gold labelled ASF antibody which will then move along the chromatographic membrane onto the test line where capture antibodies will show a wine-red line on the test cartridge (B. Zou, pers comm, 2020). A validated, accurate and affordable RDT for the confirmation of ASF could reduce the burden of testing on the laboratory during active outbreaks and provide faster decision-making tools for field staff.

The pilot nature of the study determined the sample size. The DSe and DSp of the SLB ASF RDTs are stated to be 66.7 % and 100.0 %, respectively (L. Jiangcheng, pers comm, 2019). This study used 107 samples from suspected-ASF cases from 16 provinces collected during the 2019 Lao outbreak, of which 57 were ASF positive, and 50 were ASF negative as determined by the reference ASF virus PCR using the the AgPath Mastermix and TaqMan PCR Assay (King et al., 2003). The negative samples were randomly chosen from a sampling frame extracted from the NAHL Pathogen Asset Control System (PACS) database using RStudio (RStudioTeam, 2018). The positive samples used in the study represented all available ASF-positive samples with recorded cycle threshold values (*ct* values = number of PCR cycles required to detect viral DNA) ranging from 16−38. At the time of testing the SLB RDT recommended using fresh, refrigerated whole blood stored in EDTA. Due to this study occurring after the peak of the Lao pandemic, that was not possible. The samples utilised in this study were whole blood (n = 58), hemolyzed serum (n = 36) and non- hemolyzed serum (n = 13). All test samples were stored at −80 °C prior to testing.

The SLB ASF RDT’s were performed according to the manufacturer’s instructions which are included in the appendices. The samples were thawed to room temperature before use in the RDT. All thawing and testing procedures were performed in a certificated class II biosafety cabinet. Two trained laboratory operators, blinded to the sample ASF test status, performed all testing. Briefly, four drops of each sample were mixed with a proprietary buffer in a microtube, followed by mixing within a transfer pipette eight times, before four drops of the solution were placed in the test well of the SLB ASF RDT cassette. The results were visually read at 20 min. The results of testing were stored in the PACS system and Microsoft Excel (Microsoft Corporation, USA). Raw sample results were exported to RStudio for analysis and the epi.tools() function from the epiR package was used to calculate diagnostic accuracy results and RDT characteristics (RStudioTeam, 2018; Stevenson et al., 2019). The 95 % confidence intervals for DSe and DSp were calculated using exact binomial methods. The effects of RDT result ∼ *ct* value and RDT result ∼ *ct* value + sample type were measured using binomial logistic regression after *ct* value was tested for linearity, using the glm() function in RStudio (RStudioTeam, 2018).

Examples of positive and negative RDT results using whole blood, haemolysed serum and non-haemolysed serum are presented in Fig. 1. Generally, the whole blood sample RDT results were more difficult to interpret those of serum samples due to the darkening of the test strip; however, the positive and control lines were still discernible for all sample types. The overall SLB ASF RDT accuracy of results when combining all sample types was DSe 65 %, 95 % CI [51–77] and DSp 76 %, 95 % CI [62–87] with the positive predictive value (PPV) 76 %, and the negative predictive value (NPV) 66 % (Table 1). The PCR *ct* value had a significant effect on the result for the RDT (p < 0.01) where each whole-number increase of a sample *ct* value, decreased the odds of the RDT returning a positive result by 17 %, 95 % CI [8 %–28 %]. Meaning a sample with a *ct* value of 17 had an almost 100 % probability of returning a positive value. In contrast, a sample with a *ct* value of 27.5 had a probability closer to 50 % of returning a positive RDT result (Fig. 2). The effect of *ct* value on RDT result did not vary significantly by sample type.

**Fig. 1:**
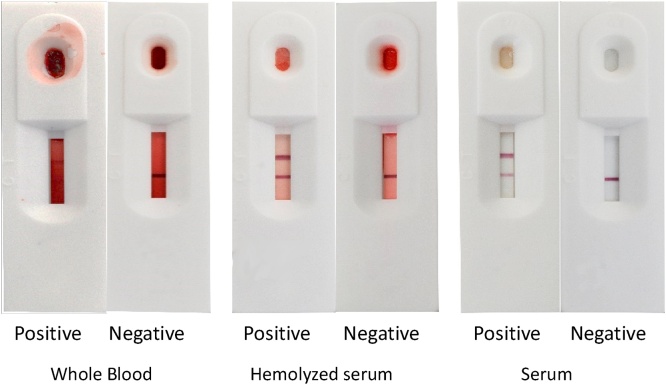
The appearance of different positive and negative sample types using the SLB ASF RDT.

**Table 1:** Diagnostic characteristics of the SLB ASF RDT using all sample types, and the performance using specific sample types.

Sample type	DSe^1^ (%) [95% CI]	DSp^2^ (%) [95% CI]	PPV^3^ (%)	NPV^4^ (%)
All samples (n = 107)	65 [51–77]	76 [62–87]	76	66
Whole blood (n = 57)	63 [44–80]	85 [66–96]	83	68
Haemolysed serum (n = 36)	50 [23–77]	68 [45–86]	50	68
Non-haemolysed serum (n = 13)	NA	83 [52–98]	91	NA

1 Diagnostic sensitivity (DSe); ^2^ Diagnostic specificity (DSp); ^3^ Positive predictive value (PPV); ^4^ Negative predictive value (NPV).

**Fig. 2:**
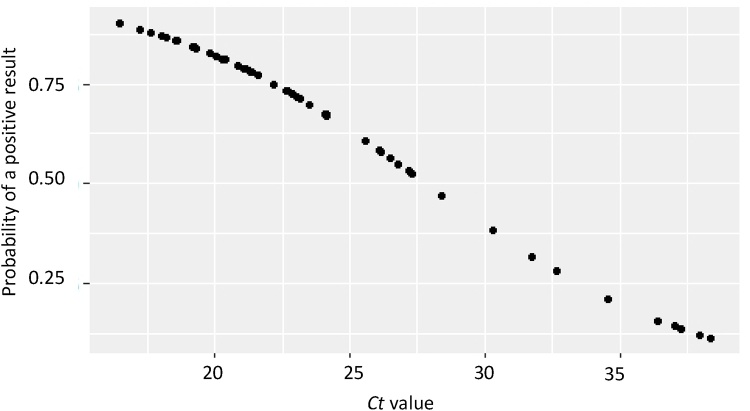
The probability of returning a positive SLB ASF RDT result given the sample's *ct* value.

This study has some limitations due to the constraints and practicalities of sample testing during the 2019 ASF outbreak. Only ASF-PCR positive samples with *ct* values and sufficient volume could be selected for this evaluation. At the height of the epidemic, the PACS system was being used to catalogue sample status. However, the sample *ct* values were not recorded in PACS until later in the year. This led to a limited number of positive samples with *ct* values being available for diagnostic evaluation in this study. Based on the RDT characteristics estimated here, the sample size used in future studies to provide 95 % confidence with a 5% margin of error should be 350 ASF-positive and 280 ASF-negative samples. The current sample size produces a 12.5 % margin of error, as reflected in the 95 % CI. The use of late epidemic samples may have introduced potential bias into the ASF-positive sample selection and reduced the number of samples available. ASF-negative samples were randomly selected from the all available PCR negative samples archived during the pandemic, which may not be representative of non-ASF diseases causing similar symptoms. Finally, data were not submitted by field veterinarians regarding symptoms or stage of the disease of each sample. This information would have provided useful information to add to the analysis, as consistent clinical signs amongst positive tests could provide a clinical syndrome to target in field testing. Future studies should aim to give helpful information to field veterinarians, such as ideal clinical presentation of animals to select for testing during an epidemic outbreak situation.

Whilst the sample test kit instructed the use of refrigerated or fresh whole blood treated with EDTA, the samples used in the study were frozen and thawed, and the use of EDTA was unrecorded. Freezing whole blood induces a level of haemolysis that cannot be avoided, and may have affected the test performance. However, the test kit instructions included a step where the sample was to be drawn up and blown out of the provided pipette, presumably to induce haemolysis in the sample. Therefore, the test kit may perform differently using fresher samples, and in future studies investigators should endeavour to utilise the SLB RDT in active outbreak situations to better determine test characteristics on fresh whole blood. Based on the initial test characteristics, it would be advisable to continue to only test anticoagulant treated whole blood samples from suspected sick or dead pigs. Focusing upon whole blood does not affect pen-side performance, as whole blood is more accessible than serum in the field.

This study demonstrates the proof of concept for the use of a low-cost RDT in the field for ASF detection in an epidemic setting where resources may be limited. The results presented here, while limited by pilot sample sizes, suggest that this test may have an application as a herd test in epidemic conditions where clinical ASF is suspected. Despite the wide confidence intervals, the results using whole blood resemble those of the similar European-produced Ingenasa test (Sastre et al., 2016). Further validation of this test with a larger sample size could provide low- and middle-income governments with a greater diversity of choice in the diagnostic market. Future studies would benefit from presenting average cost differentials between the major diagnostic categories; however, this is not within the scope of this study. We recommend that additional studies are formed to continue to define the diagnostic characteristics and applications of the SLB ASF RDT and other ASF detection rapid diagnostics that would lay the foundations to save valuable human and diagnostic resources during future ASF outbreaks.

## CRediT authorship contribution statement

**Nina Matsumoto:** Writing - original draft, Investigation, Validation, Conceptualization. **Jarunee Siengsanan-Lamont:** Project administration, Writing - review & editing, Conceptualization. **Laurence J. Gleeson:** Supervision, Writing - review & editing, Conceptualization. **Bounlom Douangngeun:** Project administration, Investigation. **Watthana Theppangna:** Investigation, Validation. **Syseng Khounsy:** Project administration, Investigation. **Phouvong Phommachanh:** Investigation, Validation. **Tariq Halasa:** Methodology, Formal analysis. **Russell D. Bush:** Supervision, Writing - review & editing. **Stuart D. Blacksell:** Supervision, Funding acquisition, Project administration, Writing - review & editing, Conceptualization.

## Declaration of Competing Interest

All authors declare that they do not have a conflict of interest.

## Footnotes

1 Av. de la Institución Libre de Enseñanza, 39 28037 Madrid, Spain.

2 Bionote Inc., 22, Samseong 1-ro 4-gil, Dongtan 1-dong, Hwaseong-si, Gyeonggi-do, Republic of Korea

3 Shenzhen Lvshiyuan Biotechnology Co., Ltd D Building, National Biological Industrial Park of Marinelife, No.2 Binhai Road, Dapeng, Shenzhen, 518120, China.
